# Henoch-Schönlein Purpura in the Adult, a Case Report

**DOI:** 10.21980/J8QH08

**Published:** 2020-01-15

**Authors:** Ivan Virovets, Danielle Biggs

**Affiliations:** * Morristown Medical Center, Department of Emergency Medicine, Morristown, NJ

## Abstract

This case report describes a dermatologic finding that is not typical in the adult population, but may be medically significant. A 39-year-old female patient with a past medical history that was negative for autoimmune disorders, vasculitides, or chronic infectious disease presented to our emergency department for evaluation of a painful rash on the bilateral lower extremities. She complained of two days of worsening raised, erythematous, pruritic and painful patches around both ankles, some of which had opened to become weeping sores. There was surrounding edema of both extremities. She denied any systemic symptoms. Online literature, primarily UpToDate was reviewed, and it was suspected that her rash may have been Henoch-Schönlein Purpura (HSP), given her lack of other medical history and a visual appearance that seemed consistent with the condition. To evaluate for renal involvement and systemic inflammation, labs were drawn and were grossly unremarkable except for a mild elevation in her C-reactive protein with a level of 37.6 mg/L. She was treated with intravenous (IV) hydration and IV ketorolac, with resultant symptomatic improvement. Since she did not have any apparent systemic involvement, she was discharged to follow up with rheumatology, with a recommendation to take over the counter non-steroidal anti-inflammatory drugs (NSAIDs). The patient was ultimately seen by rheumatology several months later, and a biopsy of the lesions demonstrated what appeared to be Henoch-Schönlein Purpura given her clinical presentation. Henoch-Schönlein Purpura can occur in adults, however rare, requiring close follow-up since in the adult population, it may result in renal failure and need for dialysis.

**Topics:**

Rash, Henoch-Schönlein purpura, dermatology, nephrology, rheumatology, vasculitis.

[Fig f1-jetem-5-1-v20]

**Figure f1-jetem-5-1-v20:**
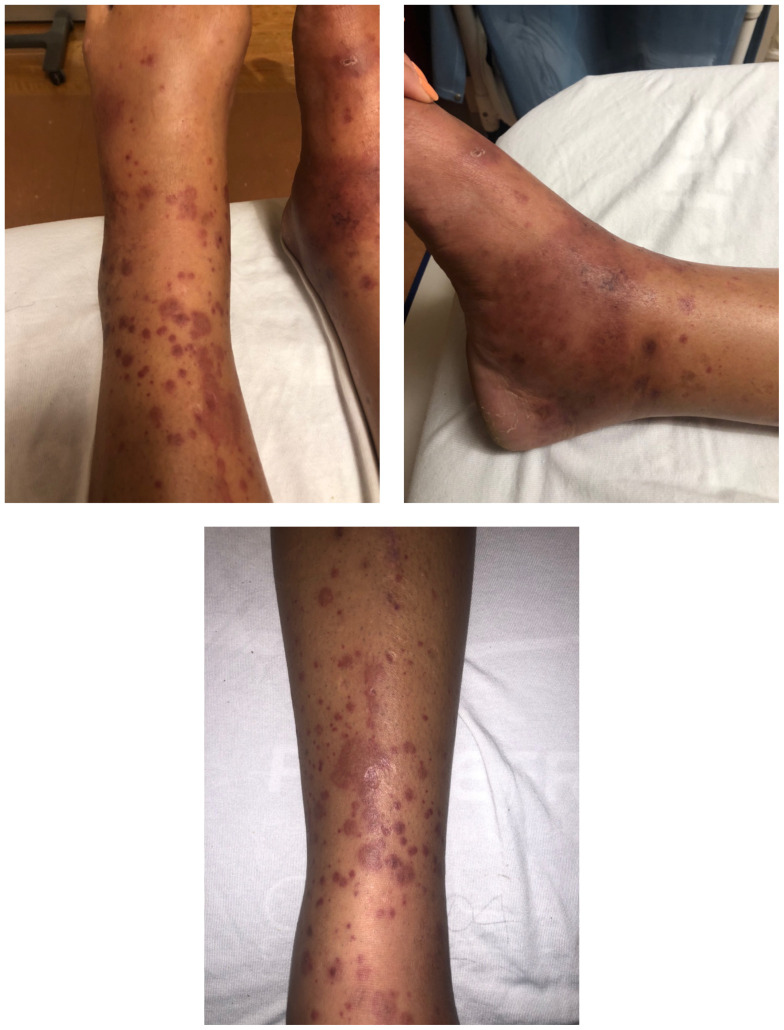


## Introduction

Patients frequently present to the emergency department (ED) with rashes of varying etiology, pathology, and significance. Frequently they are benign in nature, and respond to symptomatic treatment and self-resolve with time. However, a minority of dermatologic conditions that are seen in the ED carry medical significance and should not be missed. This adult patient presented with a rash that was suspicious for Henoch-Schönlein Purpura. This condition is rare in the adult population with an incidence of 3.4–14.3 cases per million,^1^ but carries a risk of serious renal sequelae. Studies reveal significant incidence of serious sequelae, such as renal involvement in 60% of adult patients, with 28% of them progressing to chronic renal disease.^2^ A second retrospective study by Harzallah et al, identified renal failure in 44% of patients, with 36% of those going on to develop chronic kidney disease, and an overall renal survival of 57% at 5 years.^3^ Thus, adult patients with suspected Henoch-Schönlein Purpura are at significant risk of life-altering renal injury, and so should always be referred to close specialist follow up. The rarity of Henoch-Schönlein Purpura makes this case unique, since an adult presentation is so infrequently seen.

## History of present illness

A 39-year-old female with a past medical history that was negative for autoimmune disorders, vasculitides, or chronic infectious disease, and a family history that was negative for autoimmune disease, vasculitides or connective tissue disorders presented to the emergency department with a two-day history of worsening bilateral ankle swelling, and a red patchy rash which was pruritic and painful. She was concerned that the rash was related to insect bites. Several of the patches had opened to reveal oozing sores since their appearance. She denied generalized illness, fevers, chills, abdominal pain, nausea, vomiting, or diarrhea. She denied recent gastrointestinal illness, gastrointestinal bleeding, recent upper respiratory infection, visual changes, or recent unintentional weight loss. She denies any jaundice, any other rashes, drug use, high risk sexual exposure, or any travel outside of the country or the state.

## Significant findings

The images show a raised, palpable, purpuric rash on the lower extremities, surrounded by a mild, 1+ non-pitting edema. Several of the lesions are exfoliated with serous discharge. There is no surrounding erythema, fluctuance, or lymphangitis to suggest cellulitis. There was no tenderness to palpation; however, pruritus was exacerbated on palpation.

## Patient course

The patient’s lesions were evaluated by several physicians in the ED. After review of UpToDate and literature available on the internet, the lesions were suspected to be typical of Henoch-Schönlein Purpura. Infectious causes or dermatitis were considered, but there were no physical examination findings to suggest cellulitis, and the patient did not report any new potential irritants, making this less likely. She did report suspected outdoor insect bites. However, the symmetrical and evenly distributed purpura make this an unlikely diagnosis. Drug induced vasculitis was considered; however, she denied any illicit drug use on this encounter, and was not on any medications. Systemic infectious conditions were considered, including human immunodeficiency virus and hepatitis as a cause of vasculitic lesions. However, she denied any preceding systemic symptoms with a pan-negative review of systems, and did not have any social or medical history to suggest a high risk for such conditions, having denied any history of drug use, high risk sexual contacts, or blood product exposures. Autoimmune conditions were considered; however, she had no systemic symptoms and no personal or family history of autoimmune disorders or atopy.

Laboratory studies were obtained, including a complete blood count, comprehensive metabolic panel, C-reactive protein (CRP), erythrocyte sedimentation rate (ESR), and a urinalysis. All were normal except for a mild elevation of CRP to 37.6 mg/L with a normal ESR at 22. She was treated with 15mg of IV ketorolac and 1 liter of IV normal saline for her discomfort and for renal protection, which resulted in symptomatic improvement. Guidelines as published in UpToDate, which was immediately available at the time of this encounter, were reviewed, since this condition is not commonly seen in adults, and it was determined that non-steroidal anti-inflammatory drugs for symptomatic treatment were indicated, and that steroids have not been shown to have any effect on long term gastrointestinal or renal outcome. She was subsequently discharged to home with outpatient follow-up with rheumatology.

Chart review of the period subsequent to her discharge revealed that one month after initial treatment, she was admitted for treatment of cellulitis of the affected wounds. During this admission, Henoch-Schönlein Purpura was mentioned as a differential diagnosis, but was not confirmed, and she was discharged after clinical improvement. She was seen several times by wound care since then. The following year, seven months after the initial admission, she was finally evaluated by the rheumatologist as referred, with the note citing insurance difficulties as a primary barrier to her lack of follow up. The rheumatologist noted that she had continued relapsing and remitting palpable, purpuric lesions of the same description, and considered nonspecific vasculitides, Henoch-Schönlein Purpura, autoimmune complex diseases, and chronic infectious disease such as human immunodeficiency virus, syphilis, and hepatitis as potential causes of her findings, and referred her for further testing including but not limited to antinuclear antibody, rheumatoid factor, and viral testing, which have not been performed at the time of the most recent chart review for this case. A biopsy, however, was recently performed and the histologic interpretation states “Confluent epidermal necrosis with underlying vasculitis. Considering the clinical information provided, this can represent a manifestation of Henoch-Schönlein Purpura.”

## Discussion

This case report serves as an example of a dermatologic finding that is exceedingly rare in adults, but may carry significant medical sequelae and requires close follow-up. Henoch-Schönlein Purpura most often presents in young children, and is an IgA immune-mediated vasculitis that results in abdominal pain, joint pain, and possible acute kidney injury. In young children, it is typically a self-limiting condition that results in moderate abdominal pain and the pathognomonic rash, which is typically palpable and raised, with variable degrees of skin breakdown, and generally distributed among the lower extremities. The rash may be associated with edema; however, it should be noted that edema is typically dependent, and in non-ambulatory patients such as young infants, it will be present on their backs. Severe pain may be due to intestinal involvement with purpura and may lead to intussusception by means of a mechanical lead point. In some cases, such as a case report by Lerkvaleekul et al, of a 4-year-old boy, intestinal involvement may progress to severe intestinal injury and perforation.^4^ Renal manifestations of HSP are less common in children; however, evaluation of renal function and follow-up is still warranted.

Gastrointestinal symptoms are not limited to children, albeit are less frequent in adults. Gastrointestinal involvement in adults may be severe and may require surgical management.^5^,^6^ In at least one case, in an older patient at 63 years of age, it resulted in death due to extensive bowel necrosis and hemorrhage as well as renal failure.^7^ In adults, GI manifestations are less frequent, and HSP more frequently results in development of arthritis or renal failure, in certain extreme cases requiring dialysis.^1^,^2^,^3^ Follow-up of kidney function is critical in adults.

Standard therapy in children typically consists of NSAIDs, with steroids having a questionable role in the management of this condition. Steroids have been shown to improve abdominal pain in children; however, no statistically significant effect on renal outcomes has been shown,^8^,^9^ and a 6 month review of 223 patients revealed that although steroids result in symptomatic improvement of abdominal pain in affected children, they do not alter the clinical course.^10^,^11^ A case report and literature review by Kurnia et al, confirms the utility of steroids as symptomatic therapy, without any long term benefit in terms of gastrointestinal or renal sequelae in children.^12^ In persistent cases, immunomodulators have been shown to be effective in children in chronic recurrent HSP, resulting in reduced hospitalization and improved symptoms.^13^ There is a small amount of literature regarding HSP in adults, mostly limited to case reports, and no strict guideline for therapy exists. Identification of HSP in the adult patient may also be difficult, given the higher likelihood of other forms of vasculitis and other infectious conditions, such as viral illnesses that are less common in childhood including HIV and viral hepatitis. The American College of Rheumatology 1990 guideline for identification of HSP appears to be sensitive and specific in the identification of this condition in children; however, given that one of the inclusion criteria is an age of less than 20 and presence of abdominal pain, which is less common in adults, it is less relevant in the adult patient.^14^

The main finding of this case report is the classic appearance of these lesions and their distribution in an adult, and the management and follow-up that was undertaken given these findings. The rash is classically purpuric, palpable, raised, and typically distributed in the lower extremities in the adult as it is in the child. Other vasculitides may result in palpable lesions, which may be similar to those found in this IgA vasculitis, but typically associated with systemic symptoms. It may be confused with urticarial lesions; however HSP lesions will typically be isolated to the lower extremities rather than diffuse, but will appear over a greater period of time and are associated with a greater degree of skin breakdown. Henoch-Schönlein Purpura in the adult may result in life-altering renal sequelae, even in the absence of abdominal pain, and a high index of suspicion for this condition should be maintained. The primary take-away point is that amongst numerous rashes, this one may be unexpected in an adult, should not be missed, and requires close follow-up to ensure that renal failure does not result as a complication of this condition.

## Supplementary Information






