# Supplementary figures and images for: Henoch-Schönlein Purpura in the Adult, a Case Report
Source: J Educ Teach Emerg Med. 2020 Jan 15;5(1):V20–4. doi: 10.21980/J8QH08 (PMC10332541; doi:10.21980/J8QH08)

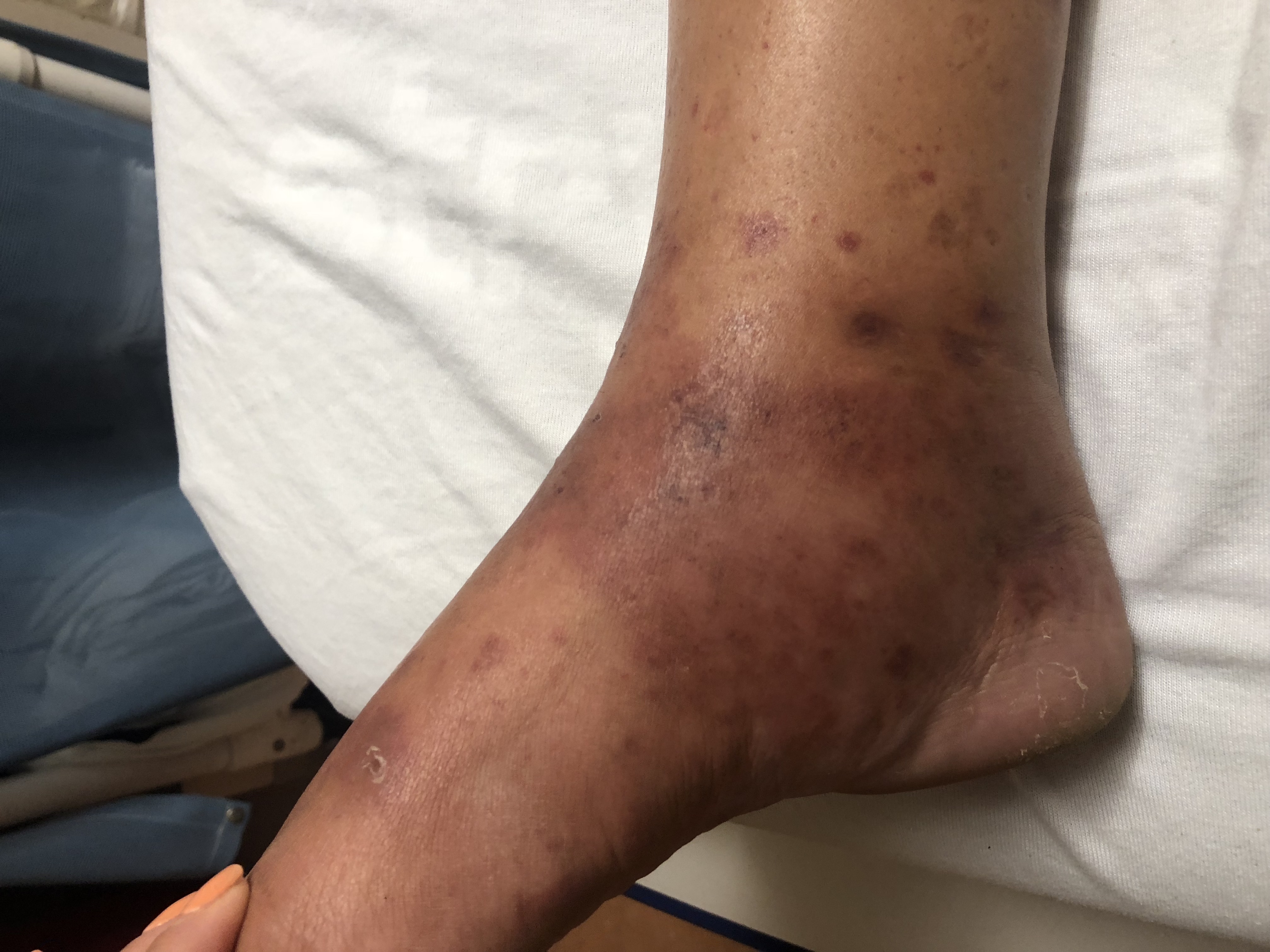

Supplement: Supplementary file 1 [file jetem-5-1-v20-supp1.jpg]

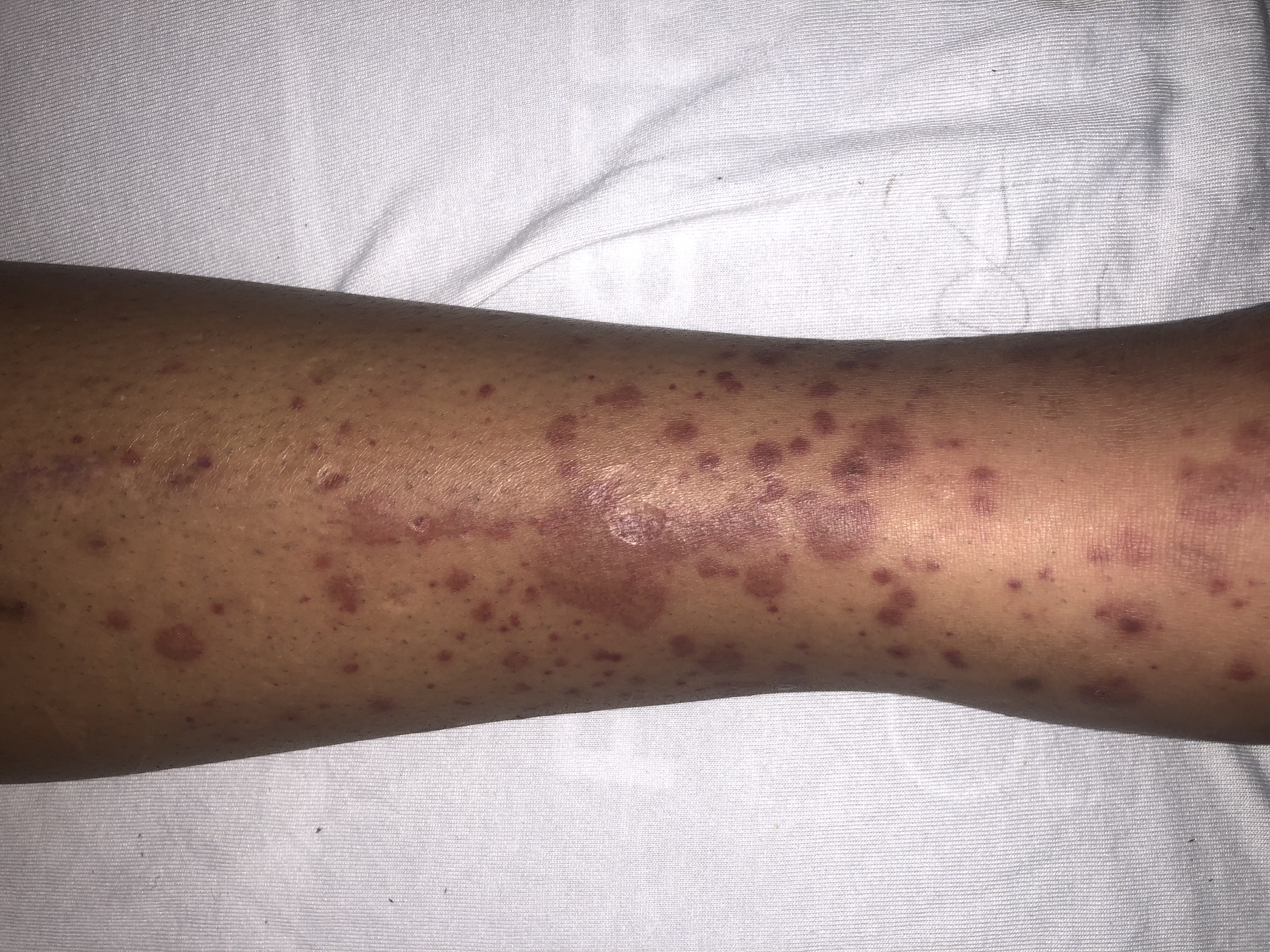

Supplement: Supplementary file 2 [file jetem-5-1-v20-supp2.jpg]

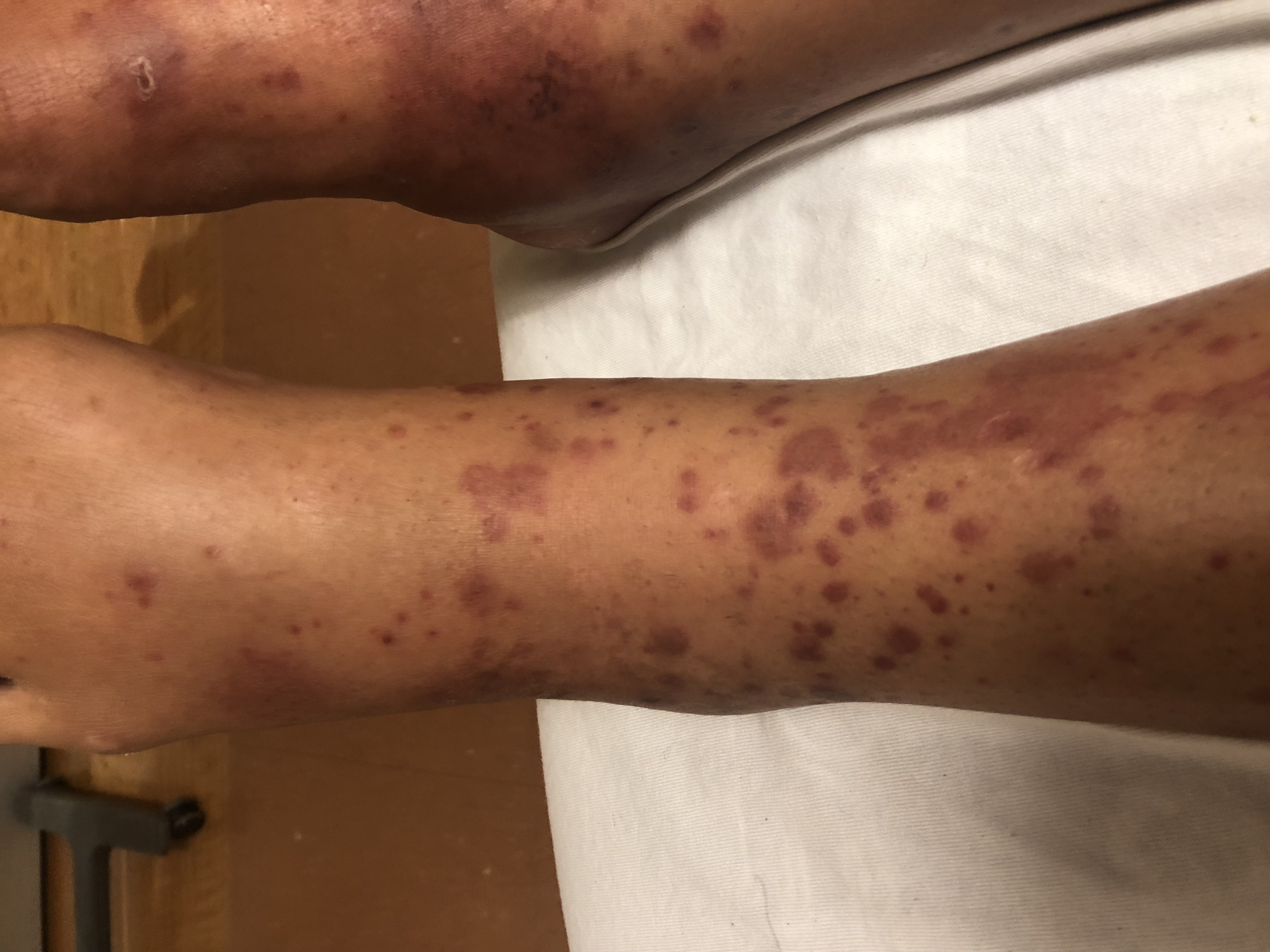

Supplement: Supplementary file 3 [file jetem-5-1-v20-supp3.jpg]
